# Combined Effects of Carbon and Nitrogen Source to Optimize Growth of Proteobacterial Methanotrophs

**DOI:** 10.3389/fmicb.2018.02239

**Published:** 2018-09-25

**Authors:** Catherine Tays, Michael T. Guarnieri, Dominic Sauvageau, Lisa Y. Stein

**Affiliations:** ^1^Department of Biological Sciences, University of Alberta, Edmonton, AB, Canada; ^2^Department of Chemical and Materials Engineering, University of Alberta, Edmonton, AB, Canada; ^3^National Renewable Energy Laboratory, Golden, CO, United States

**Keywords:** methanotrophic bacteria, methane, methanol, ammonium, nitrate, FAME

## Abstract

Methane, a potent greenhouse gas, and methanol, commonly called wood alcohol, are common by-products of modern industrial processes. They can, however, be consumed as a feedstock by bacteria known as methanotrophs, which can serve as useful vectors for biotransformation and bioproduction. Successful implementation in industrial settings relies upon efficient growth and bioconversion, and the optimization of culturing conditions for these bacteria remains an ongoing effort, complicated by the wide variety of characteristics present in the methanotroph culture collection. Here, we demonstrate the variable growth outcomes of five diverse methanotrophic strains – *Methylocystis* sp. Rockwell, *Methylocystis* sp. WRRC1, *Methylosinus trichosporium* OB3b, *Methylomicrobium album* BG8, and *Methylomonas denitrificans* FJG1 – grown on either methane or methanol, at three different concentrations, with either ammonium or nitrate provided as nitrogen source. Maximum optical density (OD), growth rate, and biomass yield were assessed for each condition. Further metabolite and fatty acid methyl ester (FAME) analyses were completed for *Methylocystis* sp. Rockwell and *M. album* BG8. The results indicate differential response to these growth conditions, with a general preference for ammonium-based growth over nitrate, except for *M. denitrificans* FJG1. Methane is also preferred by most strains, with methanol resulting in unreliable or inhibited growth in all but *M. album* BG8. Metabolite analysis points to monitoring of excreted formic acid as a potential indicator of adverse growth conditions, while the magnitude of FAME variation between conditions may point to strains with broader substrate tolerance. These findings suggest that methanotroph strains must be carefully evaluated before use in industry, both to identify optimal conditions and to ensure the strain selected is appropriate for the process of interest. Much work remains in addressing the optimization of growth strategies for these promising microorganisms since disregarding these important steps in process development could ultimately lead to inefficient or failed bioprocesses.

## Introduction

Methane-oxidizing bacteria (MOB), or methanotrophs, oxidize single-carbon molecules, specifically methane, to be used as their sole carbon and energy source. Methanotrophs are widely distributed in the environment, from rice paddies to upland soils to marine environments, among others (Bender and Conrad, 1994). Methanotrophic bacteria are taxonomically diverse and are found in the phyla Verrucomicrobiae (Dunfield et al., 2007), NC10 (Ettwig et al., 2009), and Proteobacteria (Bowman, 2006; Kelly et al., 2014; Webb et al., 2014). Within the Proteobacteria, which encompass the majority of currently cultured methanotrophs, MOB can be further classified as Alphaproteobacteria (Alpha-MOB and Type II) or Gammaproteobacteria (Gamma-MOB, Type I, or Type X), with each group having distinct physiological traits. Differentiating traits include their primary central carbon pathways (serine pathway in Alpha-MOB and ribulose monophosphate pathway in Gamma-MOB), orientation and distribution of intracytoplasmic membranes (ICMs), and composition of lipids in terms of fatty acid proportions (Hanson and Hanson, 1996).

Methane is the natural energy and carbon substrate of methanotrophs, the first molecule that is activated in their central oxidation pathway through the enzyme methane monooxygenase (MMO). MMO oxidizes methane to methanol, which is sequentially oxidized to carbon dioxide *via* formaldehyde and formate or incorporated at the level of formaldehyde into cell biomass (Hanson and Hanson, 1996). Though the pathway of methane oxidation to carbon dioxide is overall energy generating, the MMO enzyme requires energy in the form of two reducing equivalents (Hanson and Hanson, 1996). Methanotrophs can also grow exclusively on methanol and it has thus been investigated as an alternate carbon source for their culture. However, due to its toxicity, methanol as a sole growth substrate generally results in lower yields, despite the apparently decreased energetic and oxygen demands of methanol-grown cultures (Whittenbury et al., 1970; van Dijken and Harder, 1975; Best and Higgins, 1981). An exception to poor growth on methanol is the Gamma-MOB strain *Methylomicrobium buryatense* 5B, which was shown to grow faster and to higher yields when grown on methanol in batch culture (up to a concentration 1.75 M) than on methane (Eshinimaev et al., 2002). The related strain *M. buryatense* 5GB1 grew better on methane than on methanol in a bioreactor, but still demonstrated robust growth on methanol (Gilman et al., 2015), as did *Methylomicrobium alcaliphilum* 20Z (Akberdin et al., 2018).

Aside from carbon source, most methanotrophs utilize either ammonium or nitrate as nitrogen sources for assimilation while some have the capacity to fix N_2_. Theoretically, use of ammonium as a nitrogen source should be bioenergetically favorable compared to nitrate, given that it can be directly assimilated into cell biomass. However, the structural similarity between ammonium and methane leads to competitive inhibition of MMO enzymes and co-oxidation of ammonia to the cytotoxic products, hydroxylamine and nitrite (Nyerges and Stein, 2009). Toxicity and inhibition of methane oxidation by ammonium, hydroxylamine and nitrite vary significantly among methanotrophic strains (Nyerges and Stein, 2009). MOB that encode and express hydroxylamine dehydrogenase enzymes (HAO) with similarity to those found in ammonia-oxidizing bacteria can more easily overcome hydroxylamine toxicity derived from the oxidation of ammonia (Campbell et al., 2011). Yet these same strains, such as *Methylocystis* sp. Rockwell, can still be sensitive to nitrite toxicity (Nyerges et al., 2010). Then again, some methanotrophs encode and express nitrite and nitric oxide reductase enzymes that can detoxify nitrite and are thus less susceptible to these cytotoxic effects (Kits et al., 2015a; Mohammadi et al., 2017; Stein and Klotz, 2011). The presence and expression of genes for overcoming toxic intermediates of nitrogen metabolism are not phylogenetically coherent among the MOB as the ability to oxidize ammonia (i.e., nitrify) and/or reduce nitrogen oxides (i.e., denitrify) are fairly randomly distributed across MOB taxa (Stein and Klotz, 2011).

Because carbon (e.g., methane or methanol) and nitrogen (e.g., ammonium, nitrate, or N-limitation) sources have different effects on the physiology and growth of individual MOB strains, the optimization of growth medium has to be empirically determined for each isolate. For instance, a study comparing growth of the Alpha-MOB, *Methylosinus trichosporium* OB3b, and the Gamma-MOB, *Methylomicrobium album* BG8, revealed that *M. album* BG8 grew better on lower methane concentrations. Moreover, the combination of methanol and methane further enhanced growth of *M. album* BG8 over *M. trichosporium* OB3b, while *M. trichosporium* OB3b fared better than *M. album* BG8 under nitrate limitation due to its ability to fix N_2_ (Graham et al., 1993). Another study showed that the Alphas-MOB *Methylocystis* sp. Rockwell grew significantly better with ammonium, rather than nitrate, as N-source, whereas the Gamma-MOB *M. album* BG8 preferred nitrate and was uninhibited by high nitrite concentrations in the medium (Nyerges et al., 2010). A study of *Methylocystis* sp. strain SC2, showed no inhibition of growth activity with up to 30 mM ammonium, three times the standard amount in ammonium mineral salts (AMS) medium (Dam et al., 2014). Beyond growth implications, nitrogen source can also have other important implications for bioindustry. For example, nitrogen starvation serves as the most common trigger for inducing production of polyhydroxybutyrate (PHB), a carbon-based storage molecule which is a truly biodegradable polymer (Sundstrom and Criddle, 2015). Through different growth/limitation schemes, nitrogen limitation has resulted in high yields of PHB at high molecular weights; though these studies generally consider nitrogen source concentration and do not focus on nitrogen species (Khosravi-Darani et al., 2013). This is especially relevant as techno-economic analyses favor ammonium as an N-source; nitrate is a key cost driver in most bioconversion processes. As such, the growth and metabolic implications of nitrogen source are important considerations when evaluating strains for their bioindustrial potential.

The current study compares the effects of carbon source (methane or methanol) and nitrogen source (ammonium or nitrate) on growth rates and biomass yields of three Alpha-MOB and two Gamma-MOB under batch cultivation. The objectives of this study are to: (1) compare strain-to-strain variation in their carbon/nitrogen preference, (2) find preferred carbon/nitrogen combinations for each strain, and (3) determine whether changes in carbon/nitrogen sources affect the phospholipid fatty acid (PLFA) composition and/or abundance in representative strains of Alpha- and Gamma-MOB. Previous studies of strains of *M. buryatense* grown in methanol showed a significant reduction in fatty acid methyl esters (FAME) and visible reduction of ICMs (Eshinimaev et al., 2002; Gilman et al., 2015), which is logical as MMO enzymes housed in ICMs are not necessary for growth on methanol. Whether growth on methanol results in a compositional change in PLFAs remains understudied in MOB. The results of this study are useful to demonstrate the range of strain-to-strain variation in carbon/nitrogen preference among MOB toward optimized growth of strains with industrial potential.

## Materials and Methods

### Growth and Maintenance of Methanotrophic Bacteria

Five MOB isolates were selected to provide a wide comparative assessment of their growth characteristics on different carbon/nitrogen source combinations. Strains included three Alpha-MOB: *Methylocystis* sp. strain Rockwell (ATCC 49242), *Methylocystis* sp. strain WRRC1 (gift from Mango Materials), and *Methylosinus trichosporium* OB3b; and two Gamma-MOB: *Methylomicrobium album* BG8 (ATCC 33003) and *Methylomonas denitrificans* FJG1 (Kits et al., 2015b).

Cultures were grown using either AMS or nitrate mineral salts (NMS) medium (Whittenbury et al., 1970), containing either 10 mM ammonium chloride (AMS) or 10 mM potassium nitrate (NMS) as N-source. For all growth experiments, Wheaton media bottles (250 mL) closed with butyl-rubber septa caps and filled with 100 mL medium, were used as previously reported (Kits et al., 2015b). The copper (CuSO_4_) concentration in the final medium was 5 μM for all media formulations. The media were buffered to pH 6.8 through addition of 1.5 mL phosphate buffer (26 g/L KH_2_PO_4_, 33 g/L Na_2_HPO_4_) and inoculated with 1 mL (1%) of previously grown cultures that had been passaged once in identical conditions to each of the experimental conditions; as such, initial biomass at inoculation varied somewhat, reflecting the growth result of the inoculum culture.

Methane was provided *via* injection through a 0.22-μm filter-fitted syringe. 0.5, 2, or 2.5 mmol of methane were provided and the pressure was maintained at 1 atm by removing the equivalent amount of gas headspace *via* syringe prior to methane addition. To delay onset of hypoxia, the 2.5 mmol methane incubations were conducted under approximately 1.05 atm. In the appropriate experiments, 0.5, 1, or 2 mmol of pure high performance liquid chromatography (HPLC) grade methanol were added and the cultures were kept at a pressure of 1 atm. All cultures were incubated at 30°C, the optimal growth temperature for all five strains, with shaking at 150 rpm. Experiments were performed with replication (*n* = 3) for all conditions.

### Analysis of Growth

To monitor growth, 500-μL samples were extracted from cultures *via* sterile syringe at regular intervals over lag, exponential, and stationary phases. Although each growth experiment was performed multiple times to ensure consistency of growth rates and yields with each treatment, three technical replicates used for each condition to calculate standard deviations and to perform statistical analysis with even numbers of samples for each strain and condition. Growth was assessed using optical density (OD) measurements at 540 nm in a 48-well microplate (Multiskan Spectrum, Thermo Scientific). Growth rates were calculated from points on the growth curve covering an interval of logarithmic growth using the following formula (Eq. 1), where α = the growth rate constant, *N* = number of cells (herein defined by OD measurements), and *t* = time:

(1) $$\text{α}=\frac{\ln\left(\frac{N_{T}}{N_{0}}\right)}{\left(t_{T}-t_{0}\right)}$$

Growth yield was determined as the change in biomass (as measured by OD) per mole of carbon source supplied. OD was selected as a growth metric due to its widespread use in industrial bioprocess monitoring. Optimal growth conditions were chosen by weighted evaluation of both growth rate and yield, as described in Eq. 2, with the highest resultant value selected as optimal:

(2) $$x=\left(0.25\times \frac{\text{yield}}{\text{max yield}}\right)+\left(0.75\times \frac{\text{growth rate}}{\text{max growth rate}}\right)$$

Culture purity was assured through phase contrast microscopy and plating of culture on TSA/nutrient agar plates, where lack of growth demonstrated lack of contamination. Multivariate ANOVA was done using R Studio to identify contribution of factors to outcomes, as well as any interaction effects between factors.

Methane and oxygen were measured using a gas chromatograph with TCD detector (GC-TCD, Shimadzu; outfitted with a molecular sieve 5A and Hayesep Q column, Alltech). A 250-μL gas-tight syringe (SGE Analytical Science; 100 μL/injection) was used to extract and inject headspace samples. Injection and detection temperatures were 120°C and oven temperature was 90°C with current set to 90 mA, using helium carrier gas (Ultra High Purity, Praxair) at 200 kPa. Gas concentrations were calculated using standard curves of known amounts of the respective pure gases (Praxair).

### Phospholipid Fatty Acid (PLFA) Analysis

*M. album* BG8 and *Methylocystis* sp. Rockwell were selected for PLFA analysis. Cultures were grown as detailed above, with either 2.5 mmol methane or 1 mmol methanol provided as carbon source as these conditions were most favorable for biomass accumulation. Cultures were also grown with either ammonium or nitrate as N-source for comparison. Samples for analysis were collected upon reaching maximum OD_540_ but prior to the onset of stationary phase. Cells were collected by vacuum filtration onto a 0.22-μm filter, which was washed with sterile medium, at which time the cells were transferred into a microcentrifuge tube and pelleted before being frozen at -80°C. Cell pellets (*n* = 6 for each condition) were analyzed for PLFA content at the National Renewable Energy Laboratory (NREL) in Golden, CO, United States.

Whole biomass lipid content was measured through FAME analysis as described previously (Henard et al., 2016). Briefly, 10 mg of lyophyilized biomass (dried overnight at 40°C under vacuum) were homogenized with 0.2 mL of chloroform:methanol (2:1, v/v), and the resulting solubilized lipids were transesterified *in situ* with 0.3 mL of HCl:methanol (5%, v/v) for 1 h at 85 °C in the presence of a known amount of tridecanoic acid (C13) methyl ester as an internal standard. FAMEs were extracted with hexane (1 mL) at room temperature for 1 h and analyzed by gas chromatography: flame ionization detection (GC:FID) on a DB-WAX column (30 m × 0.25 mm i.d. and 0.25 μm film thickness).

### Metabolite Analysis

Supernatant (1 mL) from the same cultures used for PLFA analysis were collected *via* sterile syringe and passed through a 0.22-μm syringe filter to remove cells, with replicates grown for each condition (*n* = 3). Culture supernatants (*n* = 3), were analyzed for metabolites at the NREL in Golden, CO, United States. HPLC was used to detect lactate, formate, acetate, and methanol in culture supernatants, as described previously (Henard et al., 2016). Briefly, culture supernatant was filtered using a 0.2-μm syringe filter or 0.5 mL 10K MWCO centrifuge tube (Life Technologies) and then separated using a model 1260 HPLC (Agilent, Santa Clara, CA, United States) and a cation H HPx-87H column (Bio-Rad). A 0.1-mL injection volume was used in 0.01 N sulfuric acid with a 0.6 mL/min flow rate at 55°C. DAD detection was measured at 220nm and referenced at 360 nm, and organic acid concentrations were calculated by regression analysis compared to known standards. For analysis of comparisons between conditions, significance was determined by standard *t*-test, with α < 0.05; all differences denoted as significant met this standard.

### RNA Extraction

Total RNA was extracted from *Methylocystis* sp. Rockwell and *M. album* BG8 cells grown in either AMS or NMS, with methanol (1 mmol) or methane (2.5 mmol) provided as carbon source, at late log phase, using the MasterPure RNA purification kit (Epicentre). Briefly, cells were inactivated with phenol-stop solution (5% phenol and 95% ethanol) and pelleted through centrifugation. Nucleic acid from *Methylocystis* sp. Rockwell and *M. album BG8* were purified according to manufacturer’s instructions, with the following modifications: 1 mg total Proteinase K was added for *Methylocystis* sp. Rockwell, and 0.35 mg total Proteinase K was added for *M. album* BG8. In addition, samples of *Methylocystis* sp. Rockwell grown on methanol were processed with organic solvent extraction in place of MPC precipitation as follows: extract sequentially with equal volume of phenol (acetate-buffered, pH 4.2), equal volume of 1:1 phenol:chloroform, and equal volume of 24:1 chloroform:isoamyl alcohol, before resuming MasterPure total nucleic acid precipitation protocol at the isopropanol addition step. RNA quantity and quality were assessed using a BioAnalyzer (Agilent Technologies).

### RNA Sequencing and Assembly

RNA-Seq was performed by the Department of Energy Joint Genome Institute (DOE, JGI), using Illumina HiSeq-2000 technology. Raw reads, JGI transcriptomic analysis, and additional supporting information were made available through the JGI Genome Portal, under proposal ID 1114. Raw reads were trimmed and quality checked using CLC Genomics Workbench with quality scores (limit 0.05) and length filter (>30 bp). CLC RNASeq Assembler was then used to map reads to genome using default settings. Gene expression and differential expression were calculated using CLC Genomics Workbench, using reads per kilobase of transcript per million mapped reads (RPKM) as normalized gene expression levels. Due to its prevalence in literature, nitrate-methane was selected as the reference condition to serve as a standard of comparison, and all other expression levels were judged relative to expression under this condition. Significance in differential expression was considered at an *n*-fold change of >|1.25| and false discovery rate (FDR) adjusted *p*-value of <0.05, calculated by CLC Genomics Workbench. All *Methylocystis* sp. Rockwell conditions were completed with *n* = 3 replicates, as was *M. album* BG8 NMS/CH_3_OH, while the remaining three samples were *n* = 2 replicates.

## Results

### Effect of Carbon and Nitrogen Sources on Growth Rates and Yields of Methanotrophs

The effects of two carbon (methane and methanol) and two nitrogen (ammonium and nitrate) sources on the growth rates and yields of three Alpha-MOB and two Gamma-MOB were compared. The range of carbon amounts added to the 100-mL cultures was chosen from a point of limitation to excess as follows. At 0.5 mmol methane, the cultures were found to be carbon limited, as demonstrated by complete depletion of methane coinciding with the onset of stationary phase (**Supplementary Figure S1**). At 2.5 mmol methane, the cultures were found to be oxygen limited as the onset of stationary phase coincided with the depletion of oxygen, while methane remained in the gas headspace (**Supplementary Figure S2**). Therefore, the comparison of growth between 0.5 and 2.5 mmol carbon were selected to include growth conditions that ranged between carbon limitation and oxygen limitation.

**FIGURE 1:**
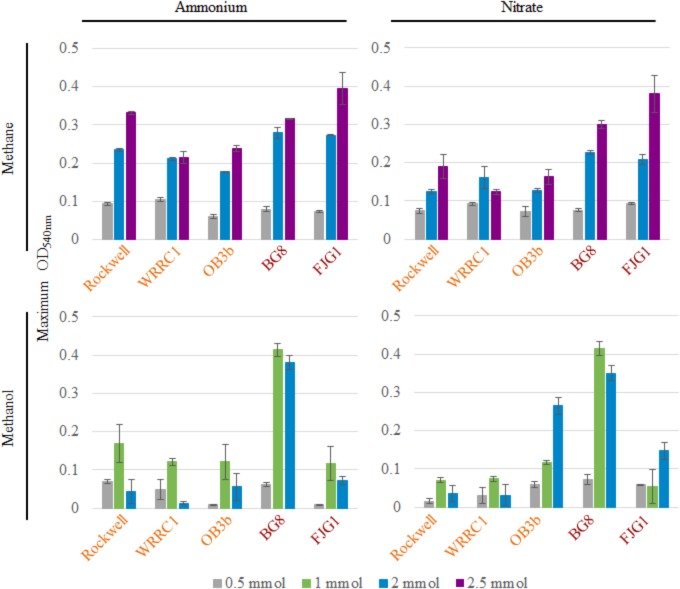
Maximum OD_540_ of 100-mL cultures of methanotrophic bacteria provided with 10 mM ammonium or nitrate and varying amounts of methane or methanol. Error bars represent standard deviations for *n* = 3 technical replicates per condition. Alpha-MOB strain names are indicated in orange and Gamma-MOB names are indicated in red.

**Figure 1** shows the maximum OD_540_ obtained for all strains and conditions tested (varying amounts of C-source, with 10 mM ammonium or nitrate in 100-mL cultures). The time points at which maximum optical densities were achieved, from the average of replicates, are given in **Supplementary Table S1**. Due to the mass transfer limitation of methane into the liquid medium, the apparent carbon availability to the culture is mediated by the surface area of the liquid–gas interface, whereas methanol is immediately available to the culture. This could lead to faster growth rates in methanol-grown cultures, as a much higher proportion of substrate is readily available for use from the time of inoculation. In some cases, the toxicity of methanol could actually result in the opposite effect, with growth inhibition occurring at higher concentrations of methanol in batch culture.

For methane-grown cultures, ammonium as the N-source resulted in overall higher biomass (OD_540_) than with nitrate, particularly for the three Alpha-MOB (**Figure 1**). At the highest methane amount tested (2.5 mmol), the two Gamma-MOB showed little difference in OD_540_ between ammonium and nitrate. In all strains, the 0.5 mmol methane condition showed low OD_540_ in agreement with the carbon limitation that this condition imposes. With nitrate, the *Methylocystis* sp. WRRC1 achieved lower OD_540_ when grown with 2.5 mmol compared to 2 mmol methane, unlike the other strains. Methanol-grown cultures generally reached a lower maximum OD_540_ than methane-grown cultures, which is apparent in both the 0.5 and 2 mmol carbon amended cultures. A notable exception to this trend was with *M. album* BG8, which showed a maximum OD_540_ when grown in 1 or 2 mmol methanol, in either ammonium or nitrate (**Figure 1** and **Supplementary Figure S3**).

Methane-grown cultures were generally more replicable in terms of growth yields (OD_540_/mol-C source) (**Table 1**) and length of lag phase (**Supplementary Table S2**) than methanol-grown cultures. Extremely low, or even absence of growth was observed among replicate cultures grown on methanol. However, higher growth yields were still achieved with 1 versus 2 mmol methanol for all strains, suggesting toxicity for 2 mmol methanol (representing a concentration of 0.2 mM). As all of the carbon was consumed in the 0.5–1 mmol carbon-amended cultures, the calculated growth yields were highest under these conditions, and were higher with methane than with methanol except for *M. album* BG8 (**Table 1**).

**Table 1:** Growth yields (OD_540nm_/mol-C source) of methanotrophic bacteria grown in combinations of different carbon and nitrogen sources.

Strain	Carbon (mmol)	Methane		Methanol	
		${\text{NH}}_{4}^{+}$	${\text{NO}}_{3}^{-}$	${\text{NH}}_{4}^{+}$	${\text{NO}}_{3}^{-}$
Rockwell	0.5	**188 (±7.98)**	149 (±13.2)	138 (±9.44)	29.7 (±13.8)
	1	–	–	169 (±50)	70.5 (±6.75)
	2	118 (±1.78)	62.2 (±2.48)	21.7 (±15.9)	17.6 (±10.7)
	2.5	133 (±1.88)	75.8 (±12.7)	–	–
WRRC1	0.5	**210 (±9.51)**	185 (±7.5)	98.3 (±51.8)	60.1 (±39.6)
	1	–	–	121 (±8.44)	73.7 (±6.4)
	2	106 (±1.69)	80.4 (±14.9)	6.17 (±1.93)	15.6 (±13.3)
	2.5	86.0 (±6.42)	49.3 (±2.89)	–	–
OB3b	0.5	123 (±10.2)	**144 (±26.8)**	19.5 (±1.71)	120 (±15.7)
	1	–	–	121 (±45.9)	116 (±5.14)
	2	89.1 (±0.671)	63.7 (±2.09)	27.9 (±17.5)	132 (±10.7)
	2.5	95.5 (±3.12)	65.1 (±7.68)	–	–
BG8	0.5	159 (±12.7)	151 (±7.56)	123 (±10.2)	144 (±26.8)
	1	–	–	414 (±17)	**415 (±17.3)**
	2	140 (±7.59)	113 (±2.79)	190 (±9)	175 (±9.67)
	2.5	127 (±0.327)	120 (±4.01)	–	–
FJG1	0.5	147 (±4.63)	**187 (±3.57)**	19.9 (±1.15)	117 (±2.27)
	1	–	–	116 (±43.3)	54.9 (±44.5)
	2	137 (±0.283)	104 (±6.65)	36.2 (±5.1)	73.5 (±11.3)
	2.5	158 (±16.5)	152 (±19.3)	–	–

*Standard deviations of three technical replicates are reported in parentheses. Bold values are the maximum yields for each strain. Dashes indicate conditions that were not examined*.

Conditions in which methane was the carbon source and ammonium was the nitrogen source resulted in generally high growth rates for all strains. Methanol led to generally slower growth than methane, with the exception of *M. album* BG8 (**Table 2**). Lag phases also tended to be much longer for growth on methanol than methane (**Supplementary Figure S3** and **Supplementary Table S2**), although the duration of lag phases for methanol-grown cultures was generally shorter for the Gamma-MOB than for the Alpha-MOB. This may be related to poorer growth in the inoculum culture or periods of adaptation to the condition and it is important to note that continuous bioprocessing operation may mitigate these impacts. Some of the strains were not able to achieve exponential growth on methanol (0.5 mmol methanol: *Methylocystis* sp. Rockwell with nitrate, *M. trichosporium* OB3b with ammonium, *M. denitrificans* FJG1 with nitrate, 2 mmol methanol: *Methylocystis* sp. WRRC1 with ammonium, *M. denitrificans* FJG1 with ammonium). Notably, an exponential phase could be measured for all strains grown in either ammonium or nitrate when provided with 1 mmol methanol, suggesting that this intermediate methanol amount (representing a concentration of 0.1 mM) was neither carbon limiting nor toxic to the cells and was the optimal concentration among the conditions tested in this study.

**Table 2:** Growth rates of methanotrophic bacteria in different combinations of carbon and nitrogen sources, reported as change in optical density (540 nm) per hour.

Strain	Carbon (mmol)	Methane		Methanol	
		${\text{NH}}_{4}^{+}$	${\text{NO}}_{3}^{-}$	${\text{NH}}_{4}^{+}$	${\text{NO}}_{3}^{-}$
Rockwell	0.5	0.112 (±0.002)	**0.116 (±0.007)**	0.0246 (±0.002)	0.0186 (±0.001)
	1	–	–	0.0389 (±0.004)	0.0144 (±0.006)
	2	0.0995 (±0.004)	0.0614 (±0.007)	0.0402 (±0.006)	0.0579 (±0.032)
	2.5	0.113 (±0.004)	0.0491 (±0.009)	–	–
WRRC1	0.5	0.111 (±0.019)	**0.128 (±0.002)**	0.0266 (±0.01)	0.0302 (±0.004)
	1	–	–	0.0570 (±0.003)	0.0263 (±0.002)
	2	0.123 (±0.004)	0.0763 (±0.008)	0.0201 (±0.003)	0.0167 (±0.001)
	2.5	0.0640 (±0.005)	0.0572 (±0.011)	–	–
OB3b	0.5	0.0778 (±0.011)	0.0594 (±0.012)	0.0146 (±0.007)	0.0393 (±0.01)
	1	–	–	0.0460 (±0.005)	0.0411 (±0.011)
	2	**0.121 (±0.009)**	0.0685 (±0.007)	0.0547 (±0.033)	0.0566 (±0.006)
	2.5	0.0811 (±0.012)	0.0497 (±0.008)	–	–
BG8	0.5	0.144 (±0.011)	0.0918 (±0.006)	0.0340 (±0.004)	0.0383 (±0.001)
	1	–	–	**0.144 (±0.044)**	0.131 (±0.05)
	2	0.130 (±0.033)	0.0978 (±0.023)	0.0551 (±0.016)	0.0471 (±0.009)
	2.5	0.119 (±0.039)	0.101 (±0.053)	–	–
FJG1	0.5	0.0856 (±0.016)	0.110 (±0.007)	0.0224 (±0.007)	0.0596 (±0.004)
	1	–	–	0.127 (±0.008)	0.107 (±0.037)
	2	0.164 (±0.005)	0.129 (±0.008)	0.0590 (±0.025)	0.0648 (±0.01)
	2.5	**0.289 (±0.07)**	0.188 (±0.034)	–	–

*Standard deviations of three technical replicates are reported in parentheses. Bold values are the maximum growth rates during exponential phase for each strain. Dashes indicate conditions that were not examined*.

While clearly distinct growth outcomes can be noted, multivariate ANOVA analysis was completed to distinguish how strain type, carbon amount, carbon source, and nitrogen source alone and in combination contributed to maximum OD, growth rate, and growth yield for each strain (**Table 3**). All factors and combinations had statistically significant effects on maximum OD. Growth rate was also significantly impacted by each individual major factor, as well as by a variety of combinatorial factors. Growth yield was least affected by the analyzed factors though strain, carbon amount, and carbon type all had significant effects.

**Table 3:** Multifactorial analysis of variance (ANOVA) on measurements of maximum optical density, growth rate, and yield for each condition tested.

	Maximum OD	Growth rate	Yield
Strain	**<2.00 × 10^-16^**	**<2.00 × 10^-16^**	**7.83 × 10^-5^**
CAmt	**<2.00 × 10^-16^**	**9.87 × 10^-12^**	**6.91 × 10^-13^**
Carbon	**<2.00 × 10^-16^**	**<2.00 × 10^-16^**	**8.40 × 10^-13^**
Nitrogen	**3.10 × 10^-7^**	**3.97 × 10^-5^**	3.02 × 10**^-^**^1^
Strain:CAmt	**<2.00 × 10^-16^**	**<2.00 × 10^-16^**	**5.67 × 10^-3^**
Strain:carbon	**<2.00 × 10^-16^**	**2.57 × 10^-2^**	1.36 × 10**^-^**^1^
CAmt:carbon	**7.47 × 10^-3^**	1.48 × 10**^-^**^1^	**5.52 × 10^-6^**
Strain:nitrogen	**5.67 × 10^-12^**	9.45 × 10**^-^**^1^	4.28 × 10**^-^**^1^
CAmt:nitrogen	**9.76 × 10^-10^**	**3.17 × 10^-4^**	3.58 × 10**^-^**^1^
Carbon:nitrogen	**1.36 × 10^-10^**	**7.49 × 10^-4^**	1.67 × 10**^-^**^1^
Strain:CAmt:carbon	**3.84 × 10^-15^**	**3.95 × 10^-2^**	7.64 × 10**^-^**^1^
Strain:CAmt:nitrogen	**2.06 × 10^-4^**	**3.01 × 10^-2^**	9.57 × 10**^-^**^1^
Strain:carbon:nitrogen	**3.21 × 10^-5^**	7.55 × 10**^-^**^1^	6.30 × 10**^-^**^1^
CAmt:carbon:nitrogen	**1.27 × 10^-8^**	1.50 × 10**^-^**^1^	7.26 × 10**^-^**^1^
Strain:CAmt:carbon:nitrogen	**7.43 × 10^-3^**	4.35 × 10**^-^**^1^	7.74 × 10**^-^**^1^

*Values represent calculated *p*-values from *F*-tests. Bolded values represent those factors and combinations of factors (interactions) showing statistically significant, measureable effects on the outcome assessed at α = 0.05*.

Analysis of gene expression of the central methane oxidation pathway showed no notable difference in expression of MMO genes for *Methylocystis* sp. Rockwell grown on methane with either nitrate or ammonium despite the observed differences in growth (**Figure 1** and **Supplementary Table S3**). However, significant decreases in MMO gene expression levels were observed for growth of *Methylocystis* sp. Rockwell on methanol. In addition, expression of methanol dehydrogenase and formaldehyde activating protein genes were significantly decreased in methanol-ammonium grown cells (**Supplementary Table S3**). While these decreases in gene expression may point to a potential growth bottleneck, i.e., formaldehyde toxicity, changes in expression of these same genes were not observed in methanol-nitrate grown cells. In contrast to *Methylocystis* sp. Rockwell, expression of MMO genes in *M. album* BG8 increased in the methanol-ammonium, but not the methanol-nitrate, growth condition relative to the methane-nitrate growth condition (**Supplementary Tables S3, S4**). In the methanol-nitrate growth condition, genes for formaldehyde oxidation showed increased expression levels relative to the methane-nitrate control, while cells grown on ammonium with either carbon source showed no significant differences in expression of these genes.

### Effect of Carbon and Nitrogen Sources on Small Metabolites

To expand the analysis of carbon and nitrogen effects on methanotrophs, two strains, the Alpha-MOB *Methylocystis* sp. Rockwell and the Gamma-MOB *M. album* BG8, were selected for analysis of excreted metabolites, representing different types of methanotrophs as well as distinct substrate-based growth effects as measured by OD. Cultures were grown with either 1 mmol methanol or 2.5 mmol methane with either ammonium or nitrate at 10 mM. For all conditions tested – either strain with all carbon–nitrogen combinations – a significant amount of glycerol was measured (**Table 4**). Lactic acid was measurable for *Methylocystis* sp. Rockwell grown in methane-ammonium and methanol-nitrate. *Methylocystis* sp. Rockwell, but not *M. album* BG8, excreted formic acid in all cultures except when grown on methane-ammonium, with more detected in the methanol-grown cultures. Interestingly, *M. album* BG8 grown in methanol and nitrate produced small amounts of xylitol. While the origins of this sugar alcohol were not further investigated, its source could potentially be X5P-derived xylulose, which could implicate a pentose-phosphate pathway or phosphoketolase (PKT) bottleneck with implications for bioindustrial potential. RNA-Seq analysis identified no change in gene expression of PKT in this condition relative to nitrate-methane in *M. album* BG8. This condition did however show significant upregulation of formaldehyde-activating protein genes and down-regulation of formate dehydrogenase genes, which is not observed in either methane-ammonium or methanol-ammonium (**Supplementary Table S4**).

**Table 4:** Concentrations of metabolites excreted to supernatant by *Methylocystis* sp. Rockwell and *M. album* BG8 grown with different carbon and nitrogen sources reported in g/L.

Strain	Metabolite (g/L)	Methane		Methanol	
		${\text{NH}}_{4}^{+}$	${\text{NO}}_{3}^{-}$	${\text{NH}}_{4}^{+}$	${\text{NO}}_{3}^{-}$
Rockwell	Glycerol	0.311 (±0.027)	0.290 (±0.026)	0.338 (±0.053)	0.396 (±0.048)
	Lactic acid	0.039 (±0.055)	–	–	0.019 (±0.027)
	Formic acid	–	0.009 (±0.013)	0.138 (±0.005)	0.106 (±0.023)
	Xylitol	–	–	–	–
BG8	Glycerol	0.381 (±0.054)	0.371 (±0.037)	0.279 (±0.020)	0.370 (±0.110)
	Lactic acid	–	–	–	–
	Formic acid	–	–	–	–
	Xylitol	–	–	–	0.052 (±0.074)

*Methane was supplied at 2.5 mmol while methanol was supplied at 1 mmol per 100 mL of culture; respective nitrogen sources were supplied at 10 mM. Standard deviations of three technical replicates are reported in parentheses. Dashes indicate metabolites that were under the limit of detection*.

### Effect of Carbon and Nitrogen Sources on PLFA Composition and Abundance

**FIGURE 2:**
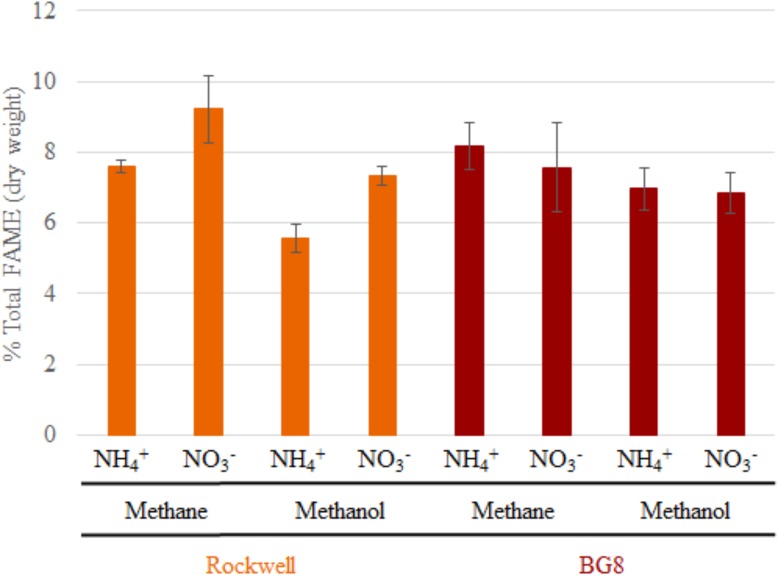
Total FAMEs measured in each sample as a percentage of total cell dry weight. Error bars represent standard deviations (*n* = 6). Results from Alpha-MOB strains are indicated in orange and from Gamma-MOB are indicated in red.

In order to determine if the combinations of carbon and nitrogen sources were significantly altering membrane structure, FAME analysis was conducted on *Methylocystis* sp. Rockwell and *M. album* BG8. All measured fatty acids were between C10 and C18, with no measurable C8 or C20–24 (which were included in the analysis standards). Overall abundance of percent biomass was determined for each strain and growth condition (**Figure 2**), and ANOVA analysis was completed to determine whether strain type, carbon type, and nitrogen type contributed to overall measured FAMEs (**Supplementary Tables S5, S6**). Total fatty acid abundance was significantly lower in methanol-grown cultures of *Methylocystis* sp. Rockwell. Furthermore, cultures of *Methylocystis* sp. Rockwell grown with ammonium had lower abundance of fatty acids than cultures grown with nitrate. In contrast, there was no significant difference in total fatty acid abundance across conditions for *M. album* BG8. Overall, strain-type, carbon and nitrogen sources and their interactions were determined to have significant impact on abundance of FAMEs (**Supplementary Table S5**).

In all conditions tested, over 93% of the fatty acid content in *Methylocystis.* sp. Rockwell was composed of only two species: C18:1n9, accounting for approximately 70–75% of the measured FAMEs, and C18:1n7, accounting for approximately 18–25% of the total FAMEs (**Supplementary Table S7**). All other fatty acids measured individually contributed less than 1.55% of the measured FAMEs. By contrast, the profile of *M. album* BG8 showed four different fatty acids contributing a substantial portion (12% or higher) of the total FAMEs measured. In descending order of prominence, these fatty acids were: C16:1n6 (36–38%), C16:1n9 (23–27%), C16:1n7 (15–20%), and C16:0 (12–15%) (**Supplementary Table S7**).

**FIGURE 3:**
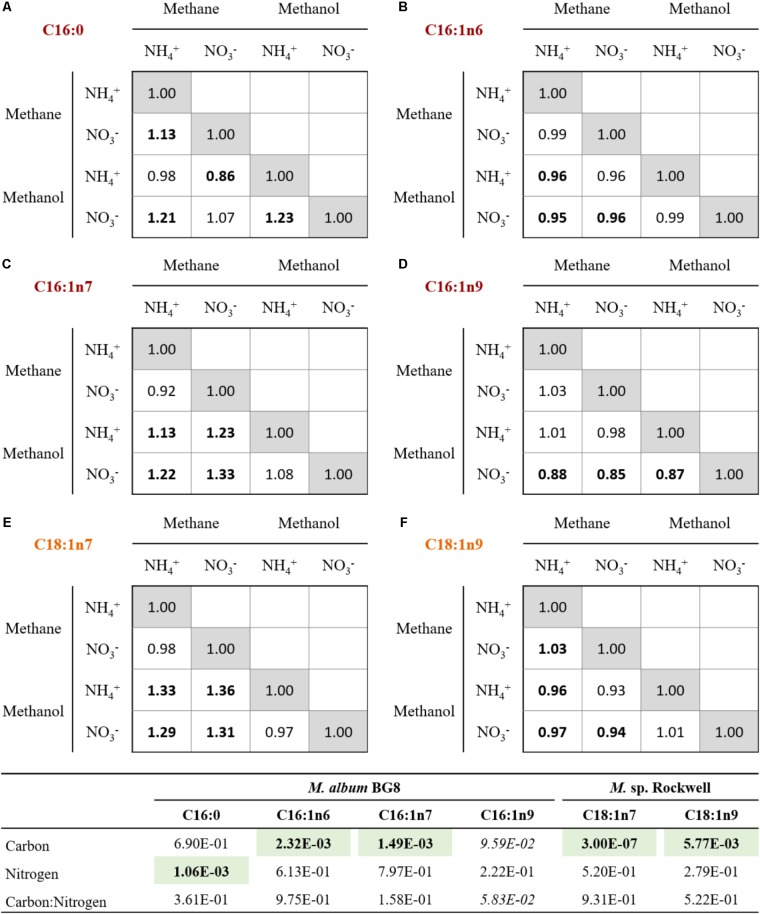
Relative changes in the abundances of primary FAMEs for cells grown with various combinations of carbon and nitrogen sources in *M. album* BG8 **(A–D)** and *Methylocystis* sp. strain Rockwell **(E,F)**. Bold values signify statistically different by unpaired *t*-test (α < 0.05).

While the general profiles held true in all cultures conditions, the relative abundance of each fatty acid varied (**Figure 3**). In *M. album* BG8, the abundance of fatty acid C16:1n6 in cells grown in methane compared to methanol was ca. 0.95:1 for both nitrogen sources. Conversely, higher proportions of the fatty acid C16:1n7 can be found in methane-fed compared to methanol-fed cultures, with differences in the abundance of this fatty acid measured at values of 1.13:1 in cells grown on ammonium and 1.33:1 in nitrate-grown cells. Both fatty acid proportions changed significantly in their response to carbon source (**Supplementary Table S6**).

Other effects of nitrogen source were noted in the C16:0 proportions, with nitrate-grown cells containing approximately 1.13 times the proportion found in ammonium-grown cells (**Supplementary Table S6**). Interestingly, the proportion of C16:1n9 was 1.12–1.15× more abundant in the methanol-nitrate condition relative to all the other conditions, though neither carbon nor nitrogen source was judged to have a significant effect.

In *Methylocystis* sp. Rockwell, a significantly lower proportion of C18:1n7 was measured as a component of total FAMEs in methanol-grown cells, with methane-grown cells possessing approximately 1.32× more C18:1n7, proportionally, regardless of nitrogen source. Carbon source likewise appeared to affect C18:1n9 composition, although conversely: methane-grown cells contained proportionally less of this fatty acid compared to methanol-grown cells, approximately 0.95:1. Both major fatty acids, C18:1n7 and C18:1n9, were significantly affected by carbon but not nitrogen source (**Supplementary Table S6**). RNA-Seq analysis of fatty acid biosynthesis pathway genes showed significantly decreased expression levels for an ACP dehydratase in *Methylocystis* sp. Rockwell grown with methanol-ammonium, and no significant change in expression under the other conditions, compared to the methane-nitrate control (**Supplementary Table S3**). In contrast, several genes in the fatty acid biosynthesis pathway in *M. album* BG8, grown only under the methanol-nitrate condition, showed both significant increase in ACP synthase gene or decreases in three genes (two ACP reductases and one ACP synthase) relative to the methane-nitrate growth condition (**Supplementary Table S4**).

## Discussion

### Optimal Carbon–Nitrogen Combinations for Growth of Methanotrophic Strains

**FIGURE 4:**
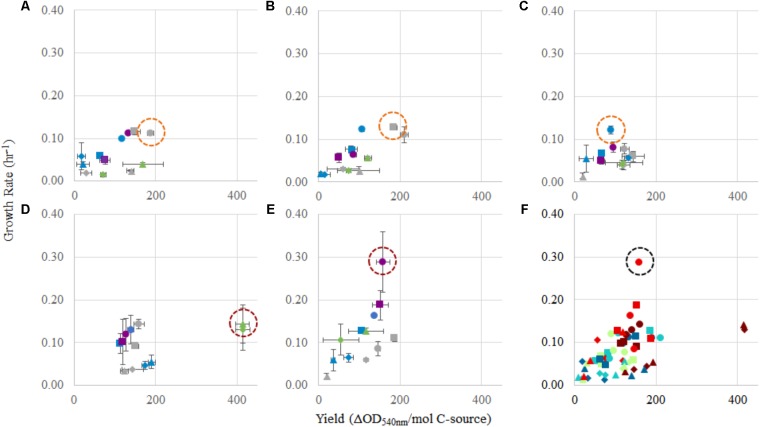
Comparison of yield (OD_540__nm_/mol C) and growth rate (h^-1^) for each strain, and in each condition tested. For panels **(A–E)**: **(A)** *Methylocystis* sp. Rockwell, **(B)** *Methylocystis* sp. WRRC1, **(C)** *M. trichosporium* OB3b, **(D)** *M. album* BG8, and **(E)** *M. denitrificans* FJG1. Carbon source amounts are: 0.5 mmol (gray), 1 mmol (green), 2 mmol (blue), and 2.5 mmol (purple). Carbon/nitrogen conditions are represented by: methane/${\text{NH}}_{4}^{+}$ (circles), methane/${\text{NO}}_{3}^{-}$ (squares), methanol/${\text{NH}}_{4}^{+}$ (triangles), and methanol/${\text{NO}}_{3}^{-}$ (diamonds). Panel **(F)** shows a combination of panels **(A–E)** together. Circles indicate best conditions for each strain **(A–E)**, or overall **(F)**.

Optimization of growth is generally approached in one of two ways, either from a maximum biomass or a fastest growth rate perspective. In an industrial context, both of these parameters have value and should be accounted for in a multi-objective optimization approach. By evaluating growth yields (**Table 1**) and growth rates (**Table 2**) together, we can determine for each strain an optimal combination of carbon–nitrogen sources, and to a lesser extent, carbon amount, leading to the best growth outcomes (**Figure 4**). The biggest limitations to these analyses are as follows: (1) lag phase was not accounted for since the use of pre-cultures and continuous cultures can overcome this limitation, (2) there is incomplete methane oxidation at higher concentrations due to O_2_ limitation (representative data in **Supplementary Figure S2**), and (3) methanol toxicity was observed at high concentrations. However, the analysis did reveal preferred combinations of carbon–nitrogen sources for each strain tested that can be further optimized to achieve the best outcomes in industrial applications.

For *Methylocystis* sp. Rockwell, methane-ammonium was the preferred carbon–nitrogen combination enabling greater yield and high growth rates, particularly for the 0.5 mmol methane amount where the carbon was completely oxidized (**Figure 4**). This condition is also most favorable for *M. trichosporium* OB3b that while achieving slightly greater yield in methane-nitrate, experienced its fastest growth rate in methane-ammonium (**Tables 1**, **2**). The optimal condition for *Methylocystis* sp. WRRC1, however, was found to be methane-nitrate at 0.5 mmol carbon source, though the weighted difference with growth rate and yield in methane-ammonium was small. In terms of industrial application, this could impact strain selection, especially when considering alternative products to biomass, fatty acids, and organic acids, as described here; previous work has found, for instance, that ammonium is a preferred nitrogen source for PHB production in *Methylocystis parvus* OBBP, but nitrate was more productive for *Methylosinus trichosporium* OB3b (Rostkowski et al., 2013). Combinatorial factors must also be considered, however, as different carbon sources may be preferred given certain nitrogen sources, or vice versa. A novel modeling-based approach has been applied to *M. trichosporium* OB3b examining such effects and demonstrates that optimal growth conditions do not match optimal PHB production conditions, and that the source of carbon, methane or methanol, changes nitrogen source preference for both metrics (Zaldívar Carrillo et al., 2018).

With these results, a balance between improved growth or product yield must be considered for *M. trichosporium* OB3b, which may not be required for *Methylocystis* sp. WRRC1 or *Methylocystis* sp. Rockwell, as PHB optimization has not yet been formally evaluated in these strains. This balance of optimization can have significant effects and must be carefully considered; use of methanol as a carbon source for production of PHB in *M. trichosporium* OB3b not only led to five times more PHB than methane but also resulted in significantly longer lag phase and delayed growth (Zaldívar Carrillo et al., 2018). Even in terms of product quality, use of methanol as a carbon source can also lead to improved molecular weight of PHB (Xin et al., 2011; Ezhov et al., 2017), but as noted in this study, may not favor optimal biomass accumulation, significantly effecting the efficiency of the overall process.

Application in a bioprocess will also necessarily consider rate and titer of the desired product, as these might dictate which substrate condition is most favorable for the particular process, including, for instance, operational mode (i.e., batch versus fed-batch, continuous etc.). Other factors than carbon and nitrogen sources must also be considered when developing an industrial process. Copper is well noted for its significance in controlling expression of pMMO and sMMO in methanotrophs (Semrau et al., 2010), and lanthanides have recently been implicated in regulating the expression of alternative methanol dehydrogenases (Farhan Ul Haque et al., 2015); neither of which were examined in this study. Nevertheless, beyond biomass, these findings may have widespread implications for diverse products, and specifically the optimized conditions for processes developed to generate these bioproducts.

While *M. album* BG8 grew favorably in most conditions tested, the 1 mmol methanol conditions proved most preferable for *M. album* BG8, with a slight preference for the methanol–ammonium combination over methanol-nitrate, largely due to the high yield resulting from these conditions. Of the five strains tested, *M. album* BG8 showed the least inhibition by substrate condition, with relatively high values resulting from analysis of weighted growth rates and yield in every experimental group. This outcome could lend well to potential future process development with this strain, given its inherent adaptability. Likely, the growth condition chosen for bioindustrial operation will need to reflect the product and process being developed; ultimately, incorporation of oxygen usage will be required to define key cost drivers and optimal process configurations. Regardless, a related industrially relevant strain, *Methylomicrobium buryatense* 5GB1, was previously found to grow faster in methane, not methanol (Gilman et al., 2015); so, this finding could point to a specialized use of *M. album* BG8 in certain industrial effluents, wherein higher concentrations of methanol can serve as a challenge for many methanotrophs.

By contrast, the best combined growth yield and rate for *M. denitrificans* FJG1 was observed with 2.5 mmol methane with either N-source suggesting efficient use of methane by this strain even under O_2_ limitation. This is interesting as this strain has an active metabolism under hypoxia, allowing for continued methane oxidation even under exceedingly low O_2_ tensions (Kits et al., 2015b), but only in nitrate, not ammonium. The growth benefit of ammonium is therefore, in this strain, unexpected. Although the lag phases for these cultures could be quite long, especially under methanol growth (**Supplementary Table S2**), the shortest lag times were observed with higher methane amounts (2.5 mmol). In an industrial process context, these data suggest that initial growth of methanotrophs could be augmented by using a higher initial methane condition before altering the carbon loading rate to achieve optimal growth yields and rates.

The excretion of particular metabolites lends clues to the efficiency of metabolism and growth of the two strains examined in more details. The accumulation of formate during growth of *Methylocystis* sp. Rockwell, particularly when grown on methanol, could imply sub-optimal conditions, and specifically an imbalance in intracellular redox potential or assimilatory bottlenecks (**Table 3**). Excretion of excess formate suggests that the C1 assimilatory pathway is not going to completion; which could explain the noticeably poorer growth outcomes, especially when growing on methanol. Decreased expression of the MMO, methanol dehydrogenase, and formaldehyde-activating protein under methanol–ammonium growth is similar to the decreased expression of genes observed for methanol growth of *M. trichosporium* OB3b (Farhan Ul Haque et al., 2017). In stark contrast, *M. album* BG8 grew robustly on methanol and even showed increased expression of genes for MMO under methanol-ammonium growth, and formaldehyde oxidation genes under methanol-nitrate growth (**Supplementary Table S4**). *M. album* BG8 also did not excrete formate (**Table 3**). Formate has been observed as an excreted metabolite during growth of other Gamma-MOB; its concentration increased as a function of unbalanced growth under oxygen limitation (Kalyuzhnaya et al., 2013) and during growth on methanol (Gilman et al., 2015), suggesting its utility as a metabolic marker for sub-optimal conditions. Production of lactate by *Methylocystis* sp. Rockwell suggests anaerobic metabolism, although this product has not been reported for other Alpha-MOB. However, *Methylocystis parvis* has been reported to produce other fermentation products like succinate and acetate during anaerobic metabolism (Vecherskaya et al., 2009). *M. album* BG8 did not excrete measurable formate into the medium under any condition, suggesting complete oxidation of methane/methanol to CO_2_ under all tested conditions.

### Carbon and Nitrogen Effects on Lipid Composition in Alpha- and Gamma-MOB

Analysis of PLFA compositions and abundances in *Methylocystis sp.* Rockwell confirmed prior studies of other *Methylocystis* sp. strains in which relative PLFA abundances, but not compositions, changed for cells grown in methane or methanol or in methane plus methanol (Bodelier et al., 2009). Overall, analysis of total fatty acids as a percentage of cell dry weight showed greater change in abundance with variation in carbon and nitrogen source in *Methylocystis* sp. Rockwell compared to *M. album* BG8 (**Figure 2**). However, both strains showed specific PLFA changes in response to different carbon and nitrogen sources (**Figure 3**). *Methylocystis* sp. Rockwell generally grew more robustly with ammonium, yet it produced significantly less PLFA than when growing with nitrate in either methane or methanol. Furthermore, methanol growth decreased the abundance of PLFA even further when compared to growth on methane. This is in agreement with previous work on *M. buryatense* 5GB1, which similarly showed a decrease in total FAMEs when grown in methanol compared to methane (Gilman et al., 2015). Overall, the FAMEs profile of *Methylocystis* sp. Rockwell, 93% composed of only two separate fatty acid types and over 75% C18:1n9, may point to suitability for use in biodiesel production, as high abundance, heavily synthesized fatty acid. The relationship between PLFA abundance and growth characteristics remains to be defined and points to an interesting area for future investigation. The PLFA abundance changes in response to carbon and nitrogen sources by *Methylocystis* sp. Rockwell is in stark contrast with the relative lack of change in *M. album* BG8.

Significant changes in gene expression of four fatty acid biosynthesis genes in *M. album* BG8 versus one in *Methylocystis* sp. Rockwell (**Supplementary Tables S3, S4**), suggests that regulation of these pathways differs dramatically between these two organisms. Expression of four fatty acid biosynthesis genes significantly changed in *M. album* BG8 when grown with methanol-nitrate even though overall abundance of fatty acids remained unchanged, suggesting a discrepancy between transcription and enzymatic activity levels. Thus, while transcriptomic analysis remains a powerful and versatile tool for informing process and culturing decisions, it also must be paired with other strategies to achieve concrete insights into pathway regulation and control.

## Conclusion

The results of this study clearly show that nutrient combinations greatly impact growth yields and rates in Alpha- and Gamma-MOB, and must be carefully considered on a strain-by-strain basis when developing bioprocessing strategies. In all cases, a multi-objective optimization approach, even rudimentary, should be considered to assess advantageous conditions for both growth yields and rates.

While a single medium may support growth of most methanotrophs (i.e., NMS and AMS), some formulations are obviously better suited to some strains rather than others. Though pathways and enzymes in these organisms may be well understood, we do not yet possess the ability to necessarily predict these optimal conditions based purely on theoretical understanding (i.e., which is calculated to be most efficient). Further work will need to be completed to address this aspect of the work, if bioindustrial optimization is to be streamlined.

These results also highlight the benefit of using certain key metabolites to evaluate nutrient effects on growth, as accumulation may point to unbalanced growth or challenging growth conditions. This has implications in understanding carbon flux, an important consideration in optimizing bioindustrial processes. These growth conditions also lead to variable FAME synthesis, helpful if the industrial process could benefit from a higher accumulation of lipids in the cell. Overall, notable differences in FAMEs response across strains are expected, which further points to strain-specific optimization (although preliminary evidence suggest that total PLFA abundance in Alpha-MOB may not be as sensitive to C- and N-sources).

While this work provides a survey of different strains growing on various combinations of carbon and nitrogen sources, many other aspects of culture optimization – including copper concentrations, phosphorous and other trace elements, and lanthanides – should also be addressed in a similar fashion. The application of these optimized conditions to common bioindustrial processes, e.g., bioreactors operating in continuous or semi-continuous modes, would also provide an interesting avenue of further study, examining efficiency through scale up and industrial applications.

## Author Contributions

CT, DS, and LS conceived the idea. CT carried out the experiments and created the figures and tables. MG supervised the FAME analysis and provided the corresponding analysis. CT, MG, DS, and LS wrote the manuscript. DS and LS supervised the work. All authors have given consent to the final version of the manuscript.

## Conflict of Interest Statement

The authors declare that the research was conducted in the absence of any commercial or financial relationships that could be construed as a potential conflict of interest.
