# Supplementary material for: Combined Effects of Carbon and Nitrogen Source to Optimize Growth of Proteobacterial Methanotrophs
Source: Front Microbiol. 2018 Sep 25;9:2239. doi: 10.3389/fmicb.2018.02239 (PMC6167414; doi:10.3389/fmicb.2018.02239)
Supplement: Supplementary file 1 [file Table_1.DOCX]

Supplementary

**Supp. Fig 1.** Representative growth curve of *Methylocystis* sp. strain Rockwell with 0.5 mmol methane and nitrogen sources provided at 10 mM concentrations. The complete depletion of methane in culture headspace coincided with the cessation of log phase. Cultures were grown in 50 mL media in sealed 250 mL Wheaton bottles. OD (540 nm) shown in blue, carbon dioxide in purple, and oxygen concentration in red.


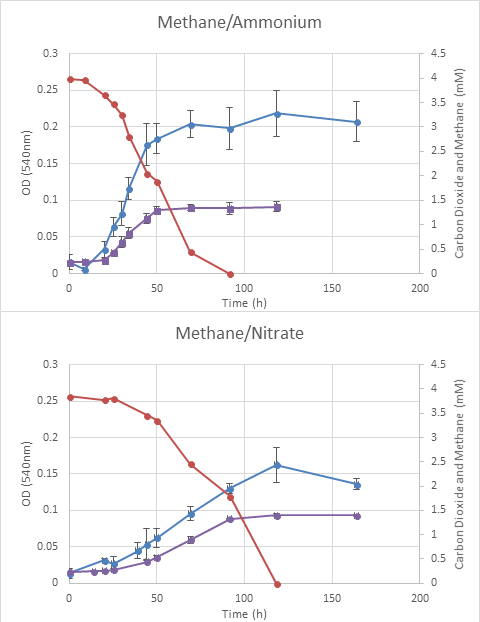


**Supp. Fig. 2.** Representative growth curves of *M. denitrificans* FJG1 showing oxygen-limited prior to carbon-limited growth. Bars represent optical density of the culture at 540 nm. Methane-ammonium growth condition is represented by blue, methane-nitrate growth condition by green. Squares represent methane concentration measured in culture headspace, circles represent oxygen concentration in headspace. Nitrogen sources were provided in 10 mM concentration, and 2.5 mmol methane was provided.

**Supp. Fig 3.** Representative growth curves of *M. album* BG8 (**a**) and *Methylocystis* sp. strain Rockwell (**b**). Methane-ammonium growth condition is represented by closed blue circles, methane-nitrate growth condition by open green circles, methanol-ammonium growth condition by closed purple triangles, and methanol-nitrate growth condition by open grey triangles. Nitrogen sources were provided in 10 mM concentration, while 2 mmol carbon sources were provided.

**
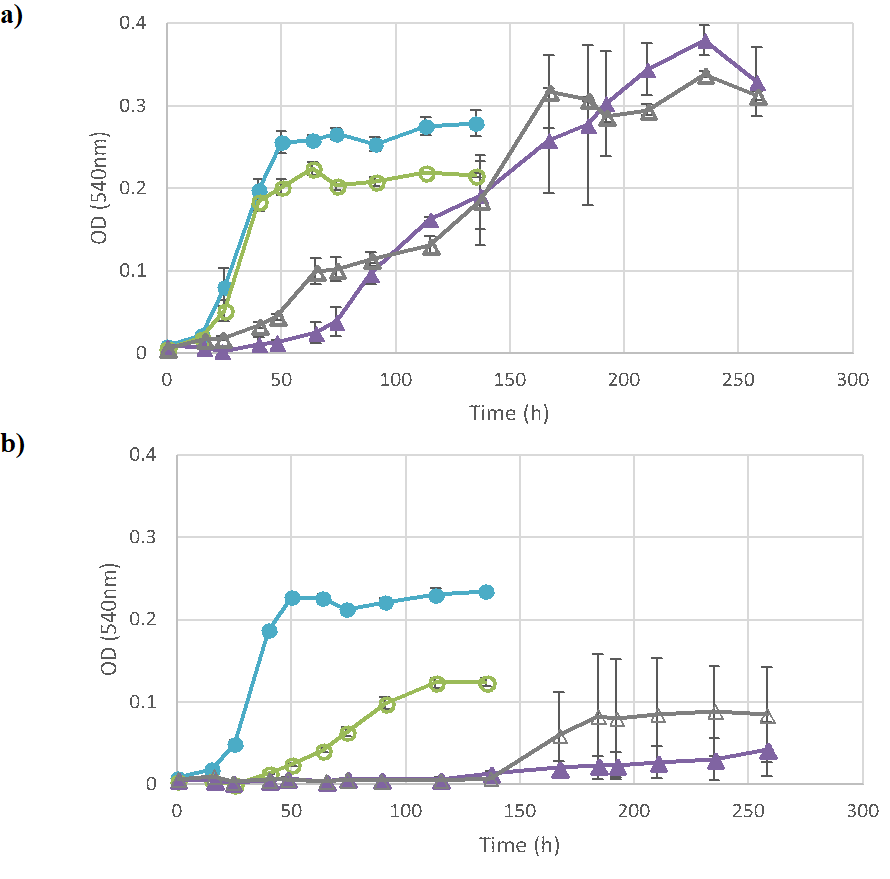
**

**Supp. Table 1.**  Time at which maximum optical density (540 nm), in hours, of methanotrophic bacteria grown in combinations of carbon and nitrogen sources in batch cultures was measured. Dashes indicate conditions that were not examined.

| **Strain** | **Carbon (mmol)** | **Methane** | | **Methanol** | |
| --- | --- | --- | --- | --- | --- |
|  |  | **NH_4_^+^** | **NO_3_^-^** | **NH_4_^+^** | **NO_3_^-^** |
| **Rockwell** | 0.5 | 187 | 84 | 208 | 231 |
|  | 1 | - | - | 168 | 168 |
|  | 2 | 135 | 135 | 258 | 235 |
|  | 2.5 | 168 | 168 | - | - |
| **WRRC1** | 0.5 | 138 | 113 | 231 | 231 |
|  | 1 | - | - | 165 | 190 |
|  | 2 | 63.5 | 113 | 235 | 258 |
|  | 2.5 | 120 | 165 | - | - |
| **OB3b** | 0.5 | 113 | 187 | 44 | 190 |
|  | 1 | - | - | 192 | 192 |
|  | 2 | 63.5 | 74 | 167 | 167 |
|  | 2.5 | 192 | 192 | - | - |
| **BG8** | 0.5 | 68 | 113 | 208 | 208 |
|  | 1 | - | - | 168 | 168 |
|  | 2 | 135 | 113 | 235 | 235 |
|  | 2.5 | 168 | 168 | - | - |
| **FJG1** | 0.5 | 68 | 44 | 70.5 | 50 |
|  | 1 | - | - | 72 | 72 |
|  | 2 | 50 | 50 | 167 | 65 |
|  | 2.5 | 68.5 | 68.5 | - | - |

**Supp. Table 2.**  Approximate lag phase, in hours, of methanotrophic bacteria grown in combinations of carbon and nitrogen sources in batch cultures. N.D. = not determined. Dashes indicate conditions that were not examined.

| **Strain** | **Carbon (mmol)** | **Methane** | | **Methanol** | |
| --- | --- | --- | --- | --- | --- |
|  |  | **NH_4_^+^** | **NO_3_^-^** | **NH_4_^+^** | **NO_3_^-^** |
| **Rockwell** | 0.5 | 15 | 15 | 50 | N.D. |
|  | 1 | - | - | 12 | 48 |
|  | 2 | 16 | 24 | 137 | 137 |
|  | 2.5 | 0 | 12 | - | - |
| **WRRC1** | 0.5 | 15 | 22 | 50 | 114.5 |
|  | 1 | - | - | 95 | 95 |
|  | 2 | 25 | 40 | N.D. | 192 |
|  | 2.5 | 0 | 25 | - | - |
| **OB3b** | 0.5 | 41 | 113 | N.D | 164 |
|  | 1 | - | - | 60 | 48 |
|  | 2 | 24 | 24 | 137 | 115 |
|  | 2.5 | 12 | 24 | - | - |
| **BG8** | 0.5 | 15 | 15 | 22 | 22 |
|  | 1 | - | - | 36 | 36 |
|  | 2 | 16 | 16 | 48 | 24 |
|  | 2.5 | 12 | 12 | - | - |
| **FJG1** | 0.5 | 22 | 15 | 22 | N.D. |
|  | 1 | - | - | 40 | 40 |
|  | 2 | 16 | 24 | N.D. | 40 |
|  | 2.5 | 40 | 24 | - | - |

**Supp. Table 3.** Differential gene expression in *Methylocystis* sp. Rockwell determined by RNA-Seq analysis, showing n-fold changes between methane-ammonium, methanol-nitrate, and methanol-ammonium growth conditions compared to the expression of genes in a methane-nitrate growth condition. Fold change was calculated from n=3 replicates for each condition. Bolded values represent those showing statistical significance (FDR-*p* < 0.05).

| **NCBI Reference Sequence** | **Gene Product** | **NH_4_^+^ /**  **CH_4_** | **NO_3_^-^ / CH_3_OH** | **NH_4_^+^ / CH_3_OH** |
| --- | --- | --- | --- | --- |
| *Methane Monooxygenase* | | | | |
| WP_036279795.1 | methane, monooxygenase, subunit C | 1.08 | -1.97 | -1.69 |
| WP_036281095.1_1 | methane, monooxygenase, subunit C | -1.27 | -1.41 | -2.02 |
| WP_036281095.1_2 | methane, monooxygenase, subunit C | 1.03 | **-2.80** | **-6.32** |
| WP_036281095.1_3 | methane, monooxygenase, subunit C | 1.14 | **-2.77** | **-6.25** |
| WP_036281738.1_1 | methane, monooxygenase, subunit A | -1.22 | **-2.12** | **-7.02** |
| WP_036281738.1_2 | methane, monooxygenase, subunit A | -1.37 | **-2.24** | **-6.94** |
| WP_036287217.1 | methane, monooxygenase, subunit B | -1.19 | -1.70 | **-5.63** |
| WP_036287347.1 | methane, monooxygenase, subunit C | -1.33 | 2.78 | 1.04 |
| WP_036288357.1 | methane, monooxygenase, subunit C | -2.50 | -1.47 | -1.97 |
| *Methanol Dehydrogenase* | | | | |
| WP_036284218.1 | methanol, dehydrogenase | -1.12 | 1.05 | 1.07 |
| WP_036285491.1 | methanol, dehydrogenase | -1.28 | -1.03 | **-3.40** |
| WP_036285493.1 | methanol, dehydrogenase | **-1.40** | 1.27 | **-4.54** |
| WP_036287664.1 | methanol, dehydrogenase | -1.04 | 1.63 | -1.27 |
| WP_036289203.1 | methanol, dehydrogenase 5 | -3.42 | -1.76 | -3.14 |
| *Formaldehyde Oxidation* | | | | |
| WP_036282879.1 | formaldehyde-activating, protein | -1.14 | 1.02 | -1.48 |
| WP_036283168.1 | aldehyde-activating, protein | 1.04 | 1.02 | -1.09 |
| WP_036288365.1 | formaldehyde-activating, protein | -1.05 | -1.06 | -2.17 |
| WP_036288367.1 | formaldehyde-activating, protein | -1.13 | 1.16 | **-3.76** |
| WP_036282881.1 | methylenetetrahydromethanopterin, dehydrogenase | 1.24 | 1.24 | 1.36 |
| WP_036284209.1 | 5-10-methenyltetrahydromethanopterin, cyclohydrolase | 1.10 | 1.09 | -1.01 |
| WP_036280414.1 | formylmethanofuran--tetrahydromethanopterin, formyltransferase | 1.46 | -1.00 | -1.22 |
| WP_036280409.1 | formylmethanofuran, dehydrogenase | 1.37 | -1.03 | 1.28 |
| WP_036280412.1 | formylmethanofuran, dehydrogenase | 1.21 | -1.04 | -1.36 |
| WP_036280416.1 | formylmethanofuran, dehydrogenase | 1.41 | -1.01 | -1.13 |
| WP_036284908.1 | methylenetetrahydrofolate, dehydrogenase | 1.47 | 1.45 | 1.44 |
| WP_036284911.1 | methenyltetrahydrofolate, cyclohydrolase | 1.47 | 1.16 | 1.23 |
| WP_036284902.1 | formate--tetrahydrofolate, ligase | 1.16 | 1.86 | 1.33 |
| *Formate Dehydrogenase* | | | | |
| WP_036282739.1 | formate, dehydrogenase | 1.42 | 1.36 | -1.12 |
| WP_036282741.1 | formate, dehydrogenase | 1.48 | 1.17 | 1.06 |
| WP_036282742.1 | formate, dehydrogenase | 1.35 | 1.49 | 1.20 |
| WP_036282743.1 | formate, dehydrogenase | 1.41 | 1.51 | 1.72 |
| WP_036287623.1 | formate, dehydrogenase | 1.49 | 1.33 | -1.07 |
| WP_036287625.1 | formate, dehydrogenase | 1.47 | 1.70 | 2.20 |
| WP_036288212.1 | formate, dehydrogenase | -2.91 | -1.44 | -3.26 |
| *Fatty Acid Biosynthesis* | | | | |
| WP_036280890.1 | acetyl-CoA, carboxylase | -1.02 | 1.26 | -1.32 |
| WP_036282352.1 | acetyl-CoA, carboxylase | -1.03 | 1.18 | -1.60 |
| WP_036282866.1 | acetyl-CoA, carboxylase, subunit, beta | 1.27 | 1.17 | 1.27 |
| WP_036286853.1 | acetyl-CoA, carboxylase | 1.29 | 1.22 | 1.81 |
| WP_036287496.1 | biotin--acetyl-CoA-carboxylase, ligase | 1.84 | -1.10 | 1.06 |
| WP_036289314.1 | acetyl-CoA, carboxylase, subunit, alpha | 1.11 | 1.03 | -1.17 |
| WP_036284556.1 | ACP, S-malonyltransferase | 1.32 | -1.07 | -1.04 |
| WP_036280951.1 | 3-oxoacyl-ACP, synthase | 1.07 | 1.09 | 1.55 |
| WP_036284276.1 | 3-oxoacyl-ACP, synthase | 1.26 | 1.15 | -1.13 |
| WP_036284547.1 | 3-oxoacyl-ACP, synthase | 1.23 | 1.06 | -1.32 |
| WP_036284553.1 | 3-oxoacyl-ACP, synthase | 1.34 | -1.00 | 1.24 |
| WP_036286470.1 | 3-oxoacyl-ACP, synthase | 1.23 | 1.01 | 1.54 |
| WP_036286640.1 | 3-oxoacyl-ACP, reductase | -2.21 | -1.34 | -5.19 |
| WP_036280295.1 | 3-hydroxyacyl-ACP, dehydratase | 1.14 | 1.02 | -1.21 |
| WP_036284278.1 | 3-hydroxydecanoyl-ACP, dehydratase | 1.15 | -1.21 | **-1.75** |
| WP_036284272.1 | enoyl-ACP, reductase | 1.19 | 1.01 | -1.02 |
| WP_036288347.1 | enoyl-ACP, reductase | 1.11 | 1.00 | -1.24 |

**Supp. Table 4.** Differential gene expression in *M. album* BG8 determined by RNA-Seq analysis, showing n-fold changes between methane-ammonium, methanol-nitrate, and methanol-ammonium growth conditions compared to the expression of genes in a methane-nitrate growth condition. Fold change was calculated from n=2 replicates for each condition, except NO_3_^-^/CH_3_OH (n=3). Bolded values represent those showing statistical significance (FDR-p < 0.05).

| **GenBank** | **Locus Tag** | **Gene Product** | **NH_4_^+^ /**  **CH_4_** | **NO_3_^-^ / CH_3_OH** | **NH_4_^+^ / CH_3_OH** |
| --- | --- | --- | --- | --- | --- |
| *Methane Monooxygenase* | | | | | |
| EIC29217.1 | Metal_1432 | methane monooxygenase/ammonia monooxygenase, subunit C | -1.00 | -1.48 | 1.10 |
| EIC29218.1 | Metal_1433 | methane monooxygenase/ammonia monooxygenase, subunit B | -1.04 | -1.65 | -1.26 |
| EIC29219.1 | Metal_1434 | Ammonia monooxygenase | -1.02 | -1.98 | -1.25 |
| EIC31238.1 | Metal_3591 | methane monooxygenase/ammonia monooxygenase, subunit B | -1.00 | -1.00 | **1.40** |
| EIC31239.1 | Metal_3592 | methane monooxygenase/ammonia monooxygenase, subunit A | -1.00 | -1.00 | **1.65** |
| EIC31240.1 | Metal_3593 | methane monooxygenase/ammonia monooxygenase, subunit C | -1.00 | -1.00 | -1.00 |
| *Methanol Dehydrogenase* | | | | | |
| EIC29181.1 | Metal_1395 | PQQ-dependent dehydrogenase, methanol/ethanol family | -1.54 | -1.10 | 1.56 |
| EIC29717.1 | Metal_1951 | beta-propeller domain-containing protein, methanol dehydrogenase | -1.20 | -2.50 | -1.66 |
| EIC30188.1 | Metal_2469 | PQQ-dependent dehydrogenase, methanol/ethanol family | 1.02 | 1.19 | 1.27 |
| EIC30191.1 | Metal_2472 | Methanol dehydrogenase beta subunit | -1.28 | 1.06 | 1.12 |
| *Formaldehyde Oxidation* | | | | | |
| EIC29284.1 | Metal_1500 | formaldehyde-activating enzyme | -1.06 | -3.84 | -1.58 |
| EIC30157.1 | Metal_2435 | formaldehyde-activating enzyme | 1.12 | **1.75** | -1.40 |
| EIC31169.1 | Metal_3521 | formaldehyde-activating enzyme | -1.11 | **2.40** | -1.00 |
| EIC28889.1 | Metal_1071 | Methylene-tetrahydromethanopterin dehydrogenase | -1.03 | 1.14 | -1.07 |
| EIC31172.1 | Metal_3524 | methenyltetrahydromethanopterin cyclohydrolase | -1.43 | **1.83** | -1.54 |
| EIC28266.1 | Metal_0412 | formylmethanofuran--tetrahydromethanopterin N-formyltransferase | -1.19 | -1.03 | 1.05 |
| EIC31166.1 | Metal_3518 | putative H4MPT-linked C1 transfer pathway protein | -1.03 | 2.20 | 1.29 |
| EIC28267.1 | Metal_0413 | formylmethanofuran dehydrogenase subunit C | -1.09 | -1.33 | 1.12 |
| EIC27971.1 | Metal_0102 | formylmethanofuran dehydrogenase subunit A | -1.07 | -1.02 | -1.17 |
| EIC28259.1 | Metal_0404 | formylmethanofuran dehydrogenase subunit B | -1.11 | 1.06 | -1.33 |
| EIC28260.1 | Metal_0405 | formylmethanofuran dehydrogenase subunit A | -1.13 | 1.08 | -1.18 |
| EIC29032.1 | Metal_1226 | flavin-dependent oxidoreductase, F420-dependent methylene-tetrahydromethanopterin reductase | 1.37 | 1.38 | 1.16 |
| EIC29186.1 | Metal_1400 | methenyl tetrahydrofolate cyclohydrolase | -1.09 | 1.07 | -1.22 |
| EIC29736.1 | Metal_1971 | formyltetrahydrofolate synthetase | -1.35 | 2.53 | -1.23 |
| *Formate Dehydrogenase* | | | | | |
| EIC29234.1 | Metal_1449 | NADH-dependent formate dehydrogenase delta subunit FdsD | 1.02 | 1.10 | 1.03 |
| EIC29235.1 | Metal_1450 | formate dehydrogenase family accessory protein FdhD | 1.07 | **-1.50** | -1.12 |
| EIC29236.1 | Metal_1451 | formate dehydrogenase, alpha subunit, archaeal-type | 1.06 | **-1.99** | -1.13 |
| EIC29203.1 | Metal_1418 | phosphoketolase | 1.09 | -1.48 | -1.48 |
| *Fatty Acid Biosynthesis* | | | | | |
| EIC28009.1 | Metal_0141 | beta-hydroxyacyl-(acyl carrier protein) dehydratase FabZ | -1.14 | 3.44 | 1.45 |
| EIC29672.1 | Metal_1906 | beta-hydroxyacyl-(acyl carrier protein) dehydratase FabA | -1.05 | -1.05 | -1.25 |
| EIC28224.1 | Metal_0368 | acyl-CoA hydrolase | -1.07 | -1.26 | -1.51 |
| EIC28378.1 | Metal_0528 | enoyl-(acyl-carrier-protein) reductase (NADH) | -1.11 | **-1.41** | -1.12 |
| EIC29396.1 | Metal_1617 | beta-ketoacyl-acyl-carrier-protein synthase II | 1.02 | 2.11 | 1.19 |
| EIC29397.1 | Metal_1618 | acyl carrier protein | 1.11 | 2.18 | 1.18 |
| EIC29398.1 | Metal_1619 | 3-oxoacyl-(acyl-carrier-protein) reductase | -1.04 | 6.91 | 1.65 |
| EIC29399.1 | Metal_1620 | malonyl CoA-acyl carrier protein transacylase | -1.07 | 6.57 | 1.55 |
| EIC29400.1 | Metal_1621 | 3-oxoacyl-(acyl-carrier-protein) synthase III | -1.15 | **5.17** | 1.30 |
| EIC29673.1 | Metal_1907 | 3-oxoacyl-(acyl-carrier-protein) synthase | -1.04 | **-1.68** | -1.09 |
| EIC30700.1 | Metal_3020 | 3-oxoacyl-(acyl-carrier-protein) synthase | -1.08 | -1.29 | -1.13 |
| EIC30701.1 | Metal_3021 | 3-oxoacyl-(acyl-carrier-protein) reductase, putative | -1.32 | -1.33 | -1.33 |
| EIC30702.1 | Metal_3022 | putative 3-hydroxylacyl-(acyl carrier protein) dehydratase | -1.13 | 1.24 | -1.07 |
| EIC30703.1 | Metal_3023 | 3-oxoacyl-(acyl-carrier-protein) synthase | -1.15 | 1.32 | 1.08 |
| EIC31544.1 | Metal_3907 | acyl-CoA dehydrogenase | 1.15 | -1.48 | -1.14 |
| EIC31546.1 | Metal_3909 | acyl-CoA dehydrogenase | 1.51 | -1.43 | -1.69 |
| EIC28009.1 | Metal_0141 | beta-hydroxyacyl-(acyl carrier protein) dehydratase FabZ | -1.14 | 3.44 | 1.45 |
| EIC29672.1 | Metal_1906 | beta-hydroxyacyl-(acyl carrier protein) dehydratase FabA | -1.05 | -1.05 | -1.25 |
| EIC28224.1 | Metal_0368 | acyl-CoA hydrolase | -1.07 | -1.26 | -1.51 |
| EIC28378.1 | Metal_0528 | enoyl-(acyl-carrier-protein) reductase (NADH) | -1.11 | **-1.41** | -1.12 |
| EIC29396.1 | Metal_1617 | beta-ketoacyl-acyl-carrier-protein synthase II | 1.02 | 2.11 | 1.19 |
| EIC29397.1 | Metal_1618 | acyl carrier protein | 1.11 | 2.18 | 1.18 |

**Supp. Table 5.** Multifactorial analysis of variance (ANOVA) on total FAMEs measured as a percent of total cell weight in *M. album* BG8 and *Methylocystis* sp. Rockwell for each condition tested. Values represent calculated F-test p-value. Bolded values represent those factors and combinations of factors (interactions) showing statistically significant, measureable effects on the outcome assessed at α=0.05.

| **Factor** |  | **FAMEs (%CDW)** |
| --- | --- | --- |
| Strain |  | **>2.00E-16** |
| Carbon |  | **>2.00E-16** |
| Nitrogen |  | **>2.00E-16** |
| Strain:Carbon |  | **3.10E-07** |
| Strain:Nitrogen |  | **>2.00E-16** |
| Carbon:Nitrogen |  | **>2.00E-16** |
| Strain:Carbon:Nitrogen |  | **7.47E-03** |

**Supp. Table 6.** Multifactorial analysis of variance (ANOVA) on specific FAMEs measured as a percent of total cell weight in *M. album* BG8 and *Methylocystis* sp. Rockwell for each condition tested. Values represent calculated F-test p-value. Bolded values represent those factors and combinations of factors (interactions) showing statistically significant, measureable effects on the outcome assessed at α=0.05. Italized values are significant at α=0.1.

|  | ***M. album* BG8** | | | | | | |  | ***M.* sp. Rockwell** | | |
| --- | --- | --- | --- | --- | --- | --- | --- | --- | --- | --- | --- |
|  | **C16:0** |  | **C16:1n6** |  | **C16:1n7** |  | **C16:1n9** |  | **C18:1n7** |  | **C18:1n9** |
| Carbon | 6.90E-01 |  | **2.32E-03** |  | **1.49E-03** |  | *9.59E-02* |  | **3.00E-07** |  | **5.77E-03** |
| Nitrogen | **1.06E-03** |  | 6.13E-01 |  | 7.97E-01 |  | 2.22E-01 |  | 5.20E-01 |  | 2.79E-01 |
| Carbon:Nitrogen | 3.61E-01 |  | 9.75E-01 |  | 1.58E-01 |  | *5.83E-02* |  | 9.31E-01 |  | 5.22E-01 |

**Supp. Table 7.** Proportion of each FAME in different carbon and nitrogen conditions in *M. album* BG8 and *Methylocystis* sp. Rockwell, as a percent of total measured FAMEs. Standard deviations of six replicates are reported in parentheses.

| **Strain** | **FAMEs** | **Methane** | | **Methanol** | |
| --- | --- | --- | --- | --- | --- |
|  |  | **NH_4_^+^** | **NO_3_^-^** | **NH_4_^+^** | **NO_3_^-^** |
| **Rockwell** | **C10:0** | 0.01 (±0.03) | 0.01 (±0.03) | 0 (±0) | 0.12 (±0.12) |
|  | **C12:0** | 0.07 (±0.01) | 0.12 (±0.09) | 0.2 (±0.05) | 0.18 (±0.06) |
|  | **C14:0** | 0.18 (±0.06) | 0.31 (±0.23) | 0.56 (±0.21) | 0.45 (±0.13) |
|  | **C15:0** | 0.05 (±0.08) | 0.13 (±0.2) | 0.2 (±0.22) | 0 (±0) |
|  | **C16:0** | 0.6 (±0.11) | 0.96 (±0.61) | 1.47 (±0.28) | 1.36 (±0.27) |
|  | **C16:1n9** | 0.24 (±0.13) | 0.59 (±0.58) | 0.23 (±0.24) | 0.65 (±0.27) |
|  | **C16:1n7** | 0.9 (±0.07) | 1 (±0.31) | 1.55 (±0.18) | 1.19 (±0.18) |
|  | **C16:1n6** | 0.07 (±0.07) | 0.08 (±0.06) | 0 (±0) | 0.04 (±0.08) |
|  | **C16:1n5** | 0 (±0) | 0 (±0) | 0 (±0) | 0 (±0) |
|  | **C16 unknown1** | 0.02 (±0.04) | 0 (±0) | 0 (±0) | 0 (±0) |
|  | **C16:2** | 0.41 (±0.08) | 0.65 (±0.48) | 0.74 (±0.55) | 1.03 (±0.4) |
|  | **C16:3** | 0 (±0) | 0.05 (±0.11) | 0.1 (±0.22) | 0.1 (±0.22) |
|  | **C18** | 0.59 (±0.12) | 0.87 (±0.54) | 1.28 (±0.27) | 1.17 (±0.24) |
|  | **C18:1n9** | 72.41 (±1.6) | 70.32 (±4.43) | 75.3 (±0.8) | 74.75 (±2.28) |
|  | **C18:1n7** | 24.45 (±1.87) | 24.9 (±2.23) | 18.36 (±0.47) | 18.95 (±2.03) |
| **BG8** | **C10:0** | 0.08 (±0.02) | 0.1 (±0.13) | 0.1 (±0.05) | 0.09 (±0.06) |
|  | **C12:0** | 0.1 (±0.02) | 0.33 (±0.08) | 0.16 (±0.03) | 0.24 (±0.05) |
|  | **C14:0** | 2.16 (±0.23) | 2.04 (±0.31) | 2.33 (±0.27) | 1.76 (±0.33) |
|  | **C15:0** | 0.58 (±0.05) | 0.67 (±0.07) | 0.46 (±0.04) | 0.55 (±0.07) |
|  | **C16:0** | 14.48 (±0.83) | 12.79 (±1.82) | 14.79 (±1.09) | 12 (±1.29) |
|  | **C16:1n9** | 24.12 (±1.73) | 23.33 (±3.05) | 23.84 (±1.76) | 27.31 (±2.67) |
|  | **C16:1n7** | 18.06 (±1.28) | 19.69 (±3.51) | 15.97 (±1.26) | 14.84 (±1.49) |
|  | **C16:1n6** | 36.59 (±0.48) | 36.81 (±1.51) | 38.16 (±0.97) | 38.41 (±0.82) |
|  | **C16:1n5** | 2.24 (±0.24) | 2.23 (±0.45) | 2.14 (±0.48) | 2.56 (±0.45) |
|  | **C16 unknown1** | 0.3 (±0.06) | 0.32 (±0.18) | 0.34 (±0.08) | 0.47 (±0.12) |
|  | **C16:2** | 0.31 (±0.08) | 0.57 (±0.5) | 0.52 (±0.04) | 0.64 (±0.18) |
|  | **C16:3** | 0.44 (±0.07) | 0.32 (±0.16) | 0.54 (±0.07) | 0.55 (±0.13) |
|  | **C18** | 0.28 (±0.06) | 0.5 (±0.28) | 0.49 (±0.08) | 0.51 (±0.09) |
|  | **C18:1n9** | 0.18 (±0.14) | 0.22 (±0.32) | 0.11 (±0.16) | 0.05 (±0.11) |
|  | **C18:1n7** | 0.08 (±0.06) | 0.1 (±0.13) | 0.03 (±0.07) | 0.03 (±0.06) |
